# Quality of adolescent and youth-friendly sexual and reproductive health services and associated factors in Ethiopia: a systematic review and meta-analysis

**DOI:** 10.3389/fpubh.2023.1191676

**Published:** 2023-07-12

**Authors:** Getachew Assefa Zenebe, Temesgen Muche Ewunie, Moges Mareg Belay, Abinet Meno Abose

**Affiliations:** ^1^School of Public Health, College of Medicine and Health Sciences, Dilla University, Dilla, Ethiopia; ^2^Department of Human Nutrition, School of Public Health, College of Medicine and Health Sciences, Dilla University, Dilla, Ethiopia; ^3^Department of Reproductive Health, School of Public Health, College of Medicine and Health Sciences, Dilla University, Dilla, Ethiopia; ^4^Department of Internal Medicine, School of Medicine, College of Medicine and Health Sciences, Dilla University, Dilla, Ethiopia

**Keywords:** quality, adolescent and youth, sexual and reproductive health services, Ethiopia, a systematic review and meta-analysis

## Abstract

**Background:**

Low-quality health care services are linked to a variety of health problems, which can have negative effects on adolescent and youth health. As a result, national data is crucial to providing high-quality healthcare to adolescents and youths in order to promote their health, wellness, and growth.

**Objective:**

To examine the quality of young people's sexual and reproductive health care services and factors associated with service satisfaction in Ethiopia.

**Methods:**

This review was carried out in accordance with the PRISMA guideline. We reviewed published data related to the quality of adolescent and youth-friendly sexual and reproductive health services (AYSRHS) in Ethiopia from January 02, 2002 to December 30, 2022. Relevant studies were identified through Google Scholar, PubMed, Cochrane Library, Science Direct, and HINARI. The extracted data was imported into STATA version 14.0 software for analysis. Heterogeneity among the reported prevalence of studies was checked using χ^2^ and I^2^ tests. The publication bias was examined by Egger's correlation and Begg's regression intercept tests at a 5% significance level.

**Results:**

The national pooled magnitude of structural, process, and output dimensions of quality of AYSRHS is 54.22% (95% CI: 33.21, 75.24%), 35.44% (95% CI: 24.95, 45.93%), and 57.01% (95% CI: 50.32, 63.7%), respectively. Being female (AOR: 1.61, 95% CI: 1.14–2.27), employed (AOR: 1.82, 95% CI: 1.06–3.14), waiting <30 min to get services (AOR: 2.7, 95% CI: 1.69–4.31), and getting information on the availability of services (AOR: 1.56, 95% CI: 1.15–2.11) were significantly associated with client satisfaction with AYSRHS.

**Conclusion:**

The overall magnitude of quality of AYSRHS in the three dimensions is far below WHO quality standards, which are 75 percent for good quality. Sex, employment status, waiting time to get services, and information on the availability of services were significantly associated with client satisfaction with AYSRHS. Therefore, different stakeholders on different levels should work together to strengthen the quality of AYSRHS concidering the above factors.

**Systematic review registration:**

Identifier [CRD42023422667].

## 1. Introduction

The World Health Organization (WHO) stated adolescents as those aged 10–19 and youths and 15–24 years, with these two age groups overlapping as “young people” in the 10–24-year age ranges (1). Young people aged 10 to 24 comprise the largest number of Ethiopians ever to enter adulthood, accounting for 33.8% of the population (2). Adolescents and youths are the creators and influencers of our global future and require particular care. However, many adolescents and youths die early, many suffer from diseases, and others may be neglected during healthcare, limiting their capacity to grow and develop to their full potential. This is due to the increased exposure of adolescents and young people to risky health behaviors (3).

Risky sexual behavior can have harmful sexual and reproductive health consequences, like unwanted pregnancy, unsafe abortion, acquired immunodeficiency syndrome (AIDS) or human immunodeficiency virus (HIV), sexually transmitted diseases (STDs), and being in a sexual relationship before being mature enough to know what constitutes a healthy relationship. People may engage in risky practices because they may not understand the concerns about HIV/AIDS and STDs, like signs and symptoms, mode of transmission, and preventive measures. Drugs and alcohol impair judgment and make unsafe sexual behavior more likely (4, 5).

To strengthen the sexual and reproductive health (SRH) of the younger generation, the WHO encourages Adolescent and Youth Friendly Reproductive Health Services (AYFRHS) and other services in different health facilities (6). Since 2006, NGOs have predominantly implemented AYFRHS programs in Ethiopia. It is now controlled by the government and deployed in existing public health institutions using an age-based strategy (7). Services that are appropriate, acceptable, and accessible to adolescents and young people are known as an adolescent- and youth-friendly services. They are offered to young people at the appropriate time, place, price, and in the proper manner. High-quality care is effective, efficient, accessible, acceptable, equitable, and safe for service users (8).

The National Adolescent and Youth Health Strategy includes nutrition, mental health, substance use, injuries, and sexual and reproductive health concerns. Additionally, the government offers thorough youth friendly services (YFS) training so that all young people in the population can be managed in a single space (age-driven approach), regardless of their complaints. This program offers a wide range of SRH services, such as family planning counseling and methods, condom promotion and distribution, testing services (for pregnancy and HIV/AIDS), management of STIs, and management of other medical diseases with appropriate referral links. In Ethiopia, YFS programs are implemented in about 44.7% of healthcare facilities using an age-driven strategy (2, 3). However, using the services is not sufficient in and of itself. It is crucial to consider the services adolescents receive because the poor quality of care is associated with a greater prevalence of sexually transmitted infections (STI) and unintended pregnancies (3).

The importance of providing high-quality health services to adolescents and youths cannot be emphasized enough. Conversely, poor health care quality is a major cause of death in a variety of diseases (9). Low-quality health care services are linked to a variety of sexual and reproductive health problems, which can have negative effects on maternal and child health. As a result, it is critical to provide adolescents and young people with high-quality healthcare in order to promote their health, wellness, and growth (1).

Adolescent and youth healthcare is still insufficient, according to research from both high- and low-income countries (1, 10, 11). The three elements of quality such as structural, process, and output dimensions and the overall magnitude of quality of services provided for the young vary by country. In Uganda 86% (12) and in South Africa lower than 89% (13) of the young people were satisfied with those services and it did not meet the declared cutoff point of 95%. Overall client satisfaction with AYFHS was lower than in a study conducted in Tanzania (14), South Africa (13) and Switzerland (15), in which 89, 87.1, and 94% of adolescents were satisfied with services, respectively. The general quality of health services provided for the young people in Ethiopia varies greatly depending on the context. For example, structural, process, and output quality dimensions were 54.4, 42.0, and 49.1%, respectively, in Southern Ethiopia (16) whereas they were 58.8, 46.4, and 47.2%, respectively, in North East Ethiopia (17). The overall quality of YFS is classified as “not good quality” or “below standard” since all of the YFS sites' performance in three of the quality dimensions fell below the predetermined cutoff criterion (75%) (16).

There are many factors contributing to the poor quality of sexual and reproductive services including socio-demographic and service-related factors. Inadequate and untrained service providers, a lack of privacy and confidentiality, negative attitudes toward providers, a lack of necessary equipment to provide the essential service package such as health information, materials, essential drugs, and supplies are the factors associated with poor service quality (1, 16–22).

Currently, Ethiopia is implementing consecutive strategic plans to improve the quality of healthcare services by taking quality and equity as one of the transformation agendas, but we haven't achieved as we expected and the agenda is continuing in the current transformation plan (23, 24).

Although studies were conducted to measure the quality of health care services provided for the young people using the Donabedian quality framework and associated factors in different parts of Ethiopia, there were discrepancies among studies observed in different settings, and that did not show a clear picture of the overall magnitude of quality in Ethiopia. There is no review of the literature on the cumulative level of health services among the young people in Ethiopia. There is a need for a study that would consider the pooled magnitude of health services provided for the young people and associated factors at the national level. In addition, knowledge of the local factors is important to understand some of the factors that influence the quality of services. This study aimed at filling this gap by examining the quality of sexual and reproductive health care services for the young people and associated factors in Ethiopia as a whole.

The information generated from this study could help the governmental institutions like Ethiopian Public Health Institution, Ethiopian Ministry of Health, and non-governmental organizations to design and implement appropriate strategies to address gaps related to the quality of those AYSRHS in public health facilities. Therefore, using a systematic review and meta-analysis, this study aimed to determine the pooled quality of healthcare services for young people and factors associated with service satisfaction in Ethiopia.

## 2. Methods and materials

### 2.1. Searching strategy

The review was carried out in accordance with the Preferred Reporting Items for Systematic Reviews and Meta-Analyses (PRISMA) guideline (25) (Supplementary material). We reviewed published and data related to the topic in Ethiopia from January 01, 2002 to December 30, 2022. Relevant studies were identified through databases (Google Scholar, PubMed, the Cochrane Library, Science Direct, HINARI, and other gray literatures). The review is registered on the prospective international register of systematic review (PROSPERO) database with the registration number CRD42023422667 (26).

The key words were combined using Boolean operators (AND/OR) to retrieve the studies: (quality OR satisfaction) AND (adolescence OR youth OR young people) AND (youth-friendly services OR reproductive health services OR sexual and reproductive health services) AND (Ethiopia).

### 2.2. Selection of studies

The titles and abstracts of retrieved studies were reviewed for relevance, and the full-text versions of potentially relevant articles were then analyzed according to the inclusion criteria detailed below. Reference lists of all included studies were checked for additional references. To avoid selection bias, the literature was searched by two authors (GAZ and TM) independently. All citations were imported into an electronic database.

### 2.3. Inclusion criteria

This review includes observational studies (cross-sectional, case-control, and cohort studies) with original data; sample sizes of more than 50 participants; participants age between 10–24 years; literature written in English; published articles and conducted in Ethiopia.

### 2.4. Exclusion criteria

Qualitative and primary studies that are not fully accessible were excluded from the study.

### 2.5. Variables and measurement

The main outcome of this study is the quality of adolescent and youth-friendly sexual and reproductive health services. It is defined as a healthcare service that satisfies a set of criteria and is evaluated on the basis of its structure (inputs), process (interaction between service providers and users), and output or level of satisfaction (27). The study has two main objectives: The first is to determine the pooled quality of adolescent and youth sexual and reproductive health services with respect to structure, process, and outcome or satisfaction dimensions in Ethiopia. The second objective is to estimate the pooled effects of each factor on the outcome quality of or satisfaction with services, and the odds ratio was calculated from the primary studies using Excel and Stata software.

Population, exposure, comparison, outcome, and study design (PECOS) were applied to the frame and answered systematic review and meta-analysis questions. Population: adolescent and youth, exposure: a determinant of satisfaction with AYSRH services, comparator: reported reference group in each included study, outcome: level of satisfaction with AYSRH services, and study design: observational study design.

### 2.6. Data extraction

Using a defined data extraction format established in Microsoft Excel, two separate authors extracted all of the required data. The author, publication year, study design, region, number of samples, quality assessment, response rate, and the magnitude of quality of services were included in the data extraction format. The data extraction format for associated factors was created in the form of a two-by-two table for each significant variable. Any differences between the authors is resolved through conversation and re-extraction of the data that is inconsistent.

### 2.7. Quality assessment

To assess the quality of the studies included in the review and meta-analysis, the Newcastle-Ottawa Scale for observational study quality evaluation was adopted (28). Using the assessment tool as a guide, two authors independently assessed the quality of the original articles. The tool's indicators are divided into three sections: the first, which includes five components, rates each study's methodological quality; the second, which examines study comparability; and the third, which assesses the original articles' statistical analysis quality. The quality of each study was evaluated by using these parameters, and articles with medium (fulfilling 50% of quality assessment criteria) and high quality were included for analysis. Assessor disagreements were resolved using the mean score of their assessment results.

### 2.8. Method of data-analysis

Important information was extracted using Microsoft Excel and then loaded into STATA version 14.0 for analysis. Text, table, and forest plot were used to describe the original articles. A heterogeneity X^2^ test and an I^2^ test were used to examine for heterogeneity among the reported magnitudes of research. At a 5% significance level, Egger's correlation and Begg's regression intercept tests were used to look for publication bias. In addition, to reduce the random variations between the point estimates of the primary studies, subgroup analysis was conducted based on the dimensions of quality, region, and publication year.

## 3. Results

### 3.1. Results of the literature search

**Figure 1:**
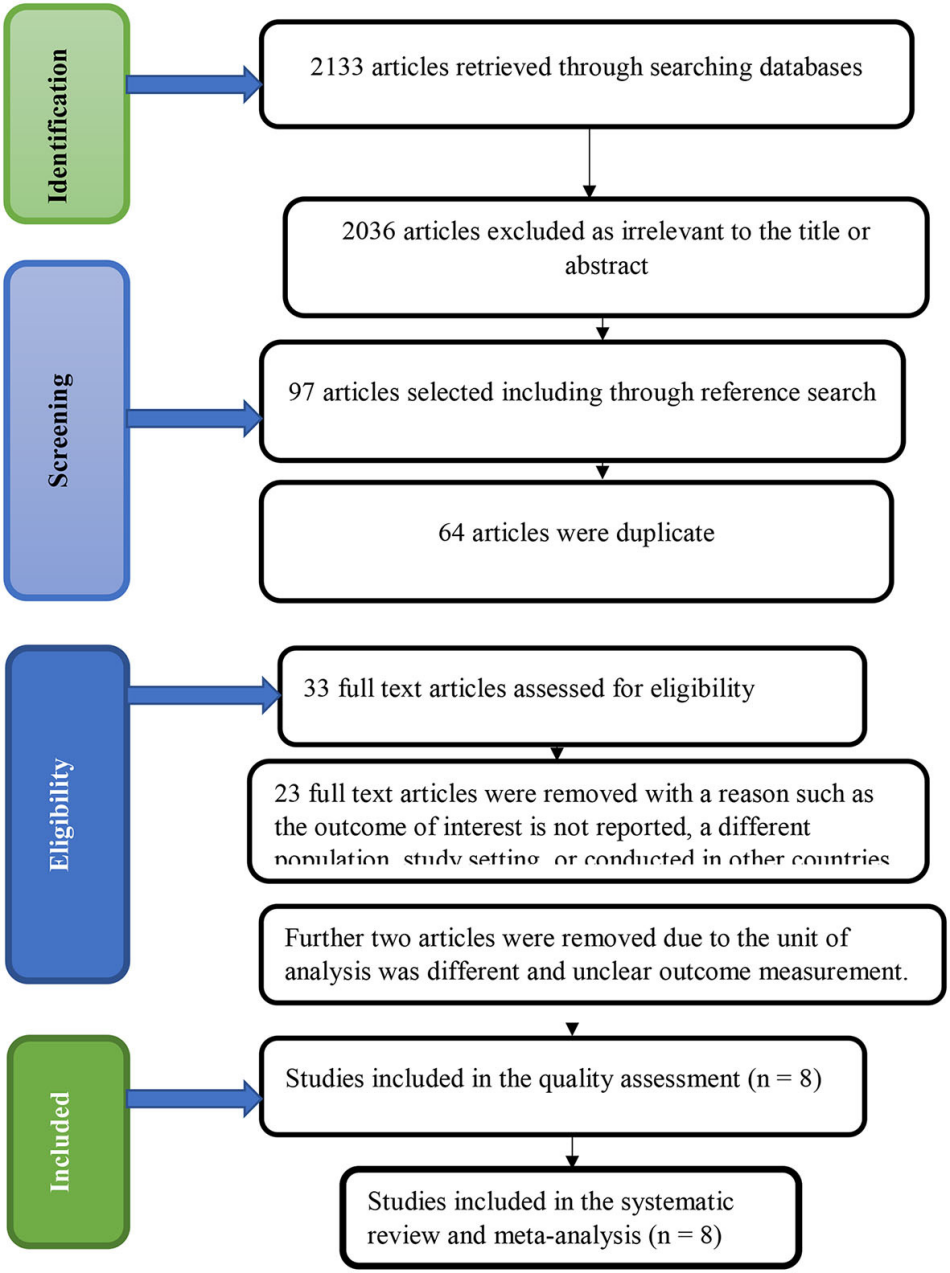
PRISMA statement presentation of systematic review and meta-analysis of quality of adolescent and youth-friendly sexual and reproductive health services in Ethiopia.

In the first step of our search, we retrieved 2,133 studies concerning the quality of adolescent and youth-friendly sexual and reproductive health services through Google Scholar, PubMed, CINHAL, Science Direct, HINARI, and other gray literature. Out of this scan, 2,036 retrieved studies were omitted via a step-by-step procedure as irrelevant to the title or abstract. Sixty-four other articles were also deleted because they were duplicates. Hence, we read the full texts of 33 articles and assessed their eligibility based on the predetermined criteria. About 23 additional studies were excluded because the outcome of interest was not reported, they involved a different population or study setting, or they were conducted in other countries. Further two articles were removed due to the unit of analysis being different from the other studies, which is the health facility (29), and the unclear outcome measurement (7). Finally, eight studies were found to be eligible and included in the systematic review and meta-analysis (Figure 1).

### 3.2. Study characteristics

Data for the eight eligible studies were extracted and analyzed in this study. Out of them, four studies assessed both the structural, process, and outcome dimensions of quality (16, 17, 30, 31), and the remaining four assessed only the outcome or satisfaction dimension of quality (32–35). To determine the pooled estimated proportion of the output dimension of quality, the client satisfaction, 4,075 youth and adolescents were surveyed. The prevalence of structural quality in adolescent- and youth-friendly sexual and reproductive health services in Ethiopia ranged from 20.6% in the Amhara region (31) to 76.6% in the Oromia region (30). The process quality aspect ranged from 28% in the Oromia region (30) to 46.4% in the Amhara region (17). In addition, the output or satisfaction aspect of quality also ranges from 47.2% in the Amhara region (17) to 70.3% in the Oromia region (30).

Of the eight studies included in this review, all of them were conducted using a cross-sectional study design. Among these, two studies were found in Oromia (30, 34), two in SNNPR (16, 33), and four in the Amhara region (17, 31, 32, 35). Regarding study settings, one study was conducted at the school level (33), two at the kebeles/household level (32, 34), and the remaining five at the health facility level (16, 17, 30, 31, 35) (Table 1).

**Table 1:** Characteristics of primary studies that assessed the quality of adolescent and youth-friendly sexual and reproductive health services in Ethiopia.

**References**	**Region**	**Study design**	**Study setting**	**Study population**	**Total sample**	**Quality assessment**	**Quality dimensions**
Mulugeta et al. (16)	Arba-Minch town, SNNP	Institution based Cross-sectional	Health centers	Young people/10–24 years age	403	9	Structure, process, output
Gebrie et al. (17)	Waghemra zone, Amhara region	Institution based Cross-sectional	Health centers	Young people/10–24 years age	431	9	Structure, process, output
Sharew et al. (35)	Dessie town, Amhara	Institution based Cross-sectional	Clinics and Health centers	Youth/15–24 years age	422	7	Output
Amenu et al. (30)	SendafaTown, Oromia region	Institution based Cross-sectional	Health centers	Young people/10–24 years age	421	8	Structure, process, output
Dagnew et al. (32)	East Gojjam Zone, Amhara	Community based Cross-sectional	Kebeles/households	Adolescents/15–19 years	313	8	Output
Habitu et al. (31)	Central Gondar Zone, Amhara	Institution based Cross-sectional	Health centers	Youth/15–24 years age	1,034	8	Structure, process, output
Helamo et al. (33)	Hadiya Zone, SNNP	Institution based Cross-sectional	Secondary schools	Youths/15–24 years age	270	7	Output
Tilahun et al. (34)	East Wollega, Oromia region	Community based Cross-sectional	Kebeles/households	Youths/15–24 years age	781	6	Output

### 3.3. Meta-analysis

#### 3.3.1. Level of structural quality of adolescent and youth-friendly health services

**Figure 2:**
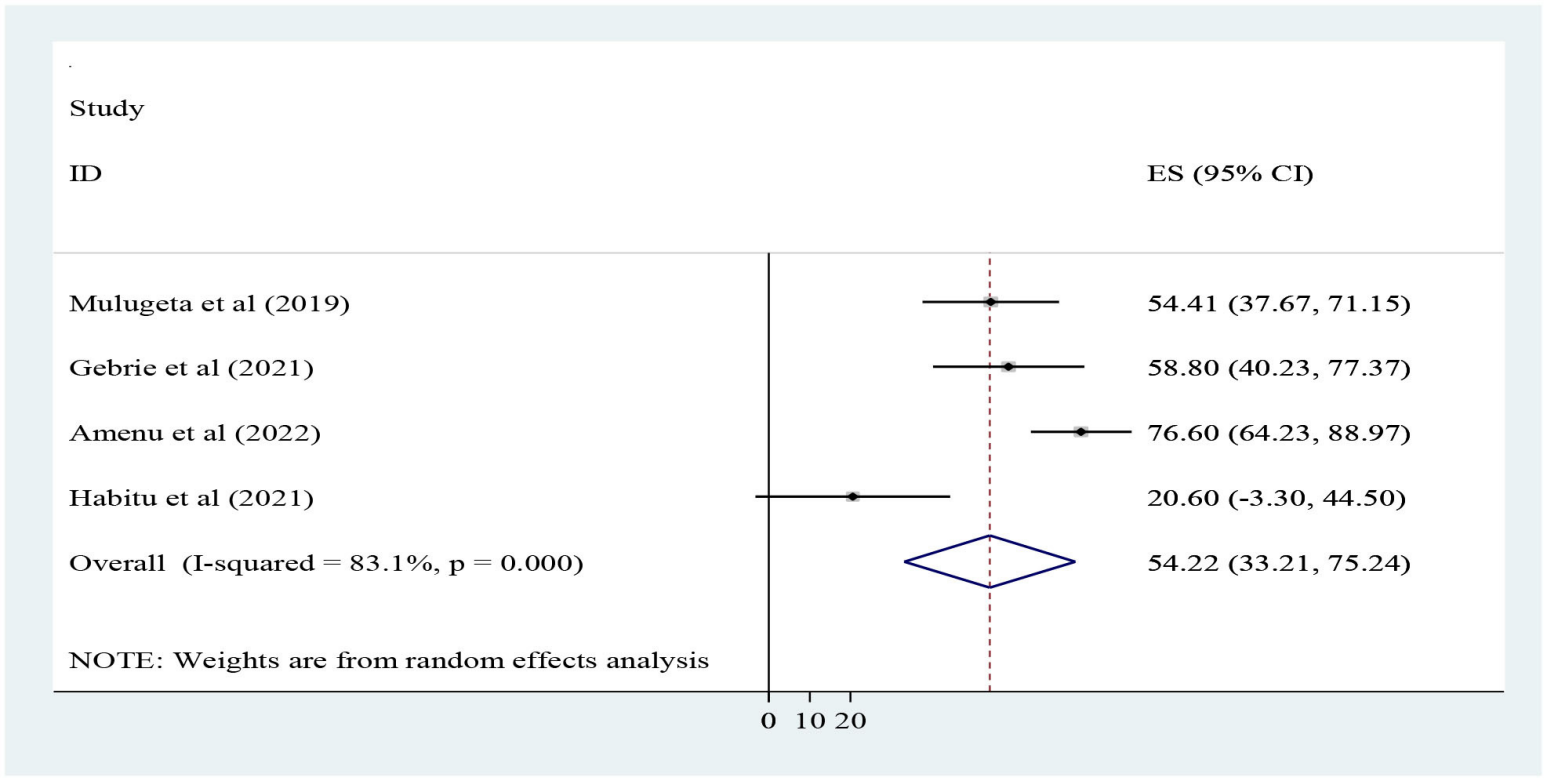
Forest plot for the pooled level of structural dimension quality of adolescent and youth-friendly health services in Ethiopia.

In the current study, the pooled magnitude of structural quality was 54.22% (95% CI; 33.21, 75.24%) with a range from 20.6 (31) to 76.6% (30). A random effect model was used, and heterogeneity was observed across the studies (I-squared = 83.1%, *p* = 0.001) (Figure 2).

#### 3.3.2. Level of process quality of adolescent and youth-friendly health services

**Figure 3:**
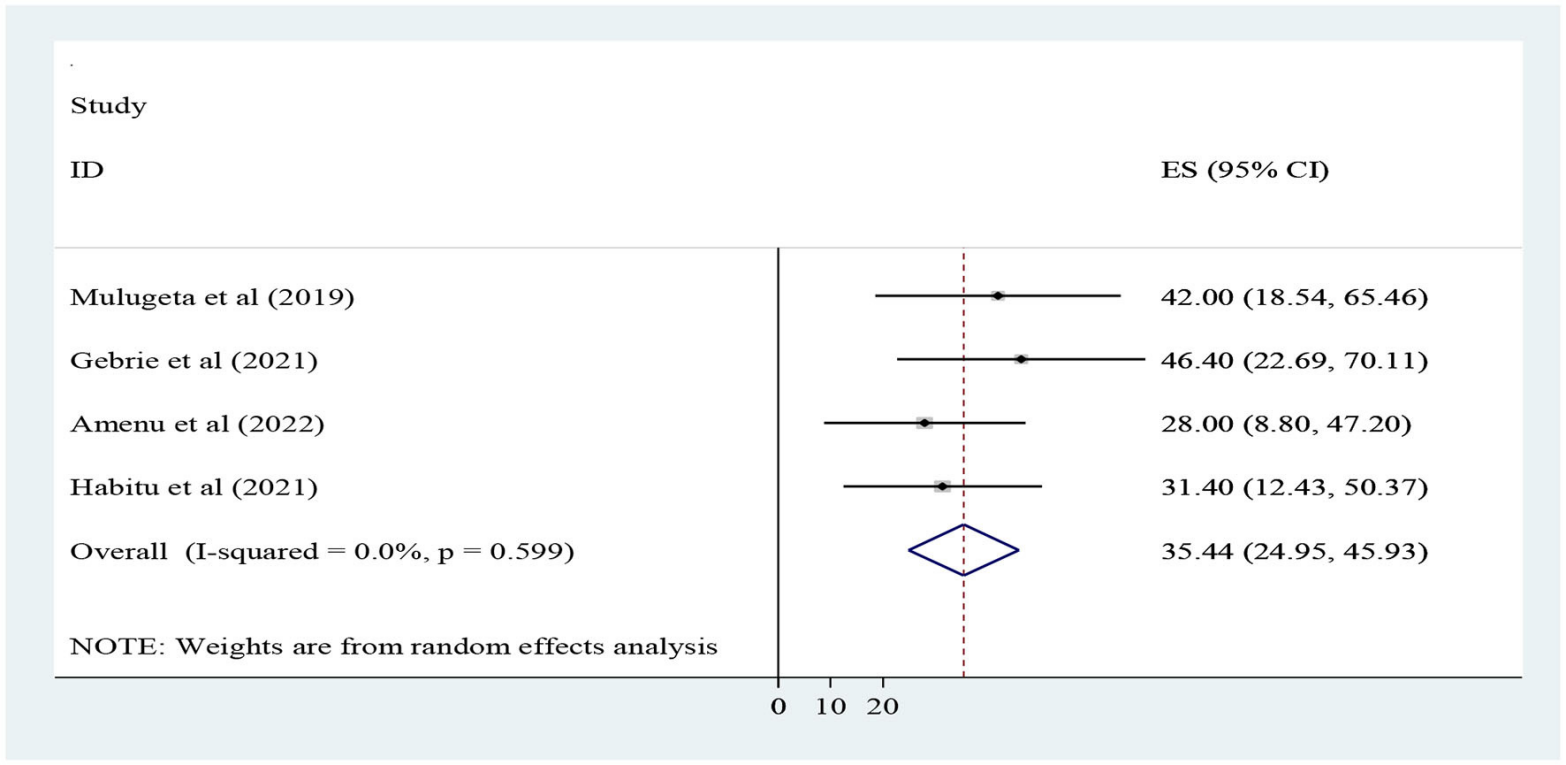
Forest plot for the pooled level of process dimension quality of adolescent and youth-friendly health services in Ethiopia.

This study shows that the pooled magnitude of process quality was 35.44% (95% CI; 24.95, 45.93%) with a range from 28 (30) to 46.4% (17). A random effect model was used, and heterogeneity was not observed across the studies (I-squared = 0.0%, *p* = 0.59) (Figure 3).

#### 3.3.3. Level of client's satisfaction on adolescent and youth-friendly health services

**Figure 4:**
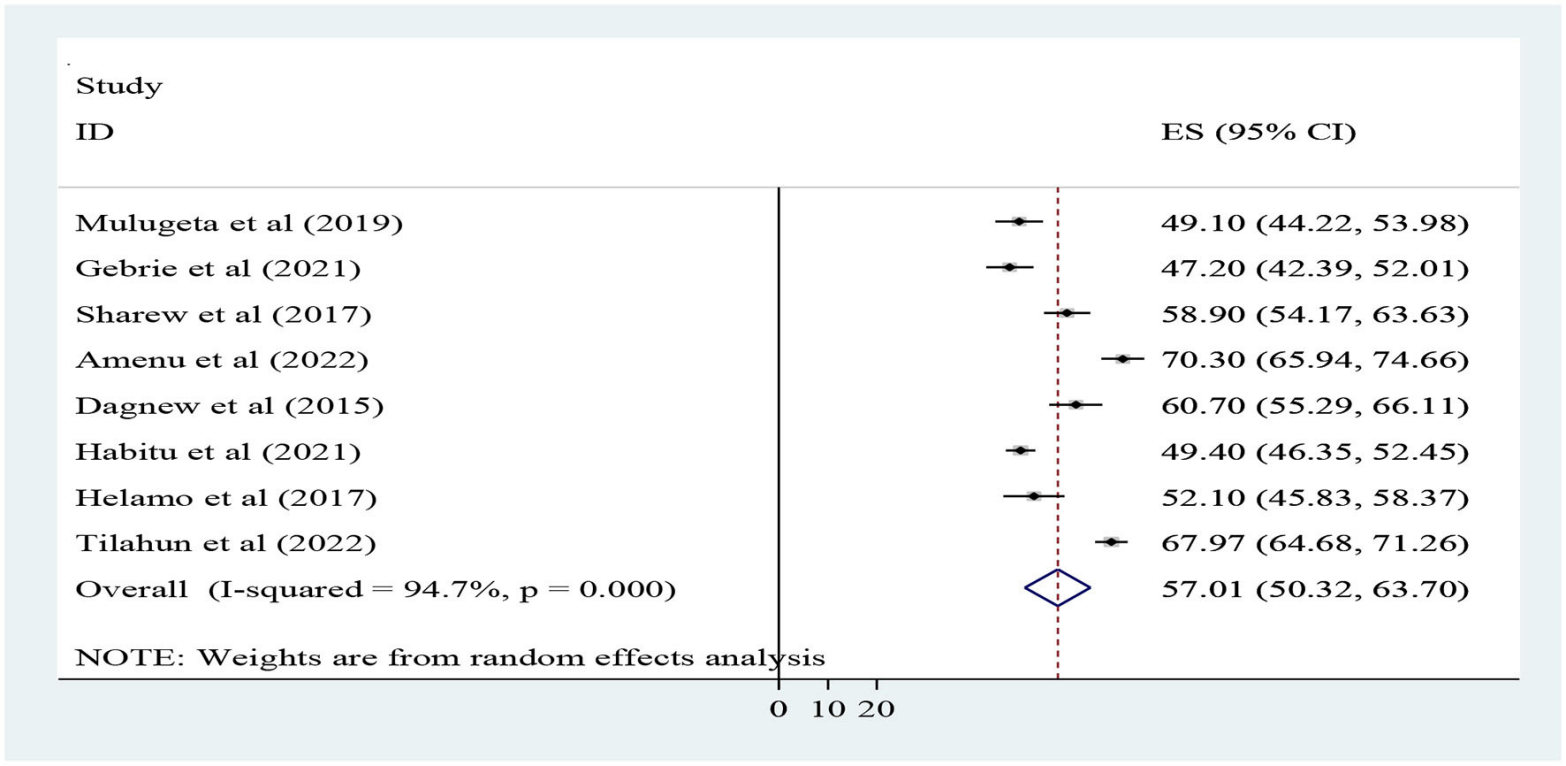
Forest plot for the pooled level of client satisfaction toward adolescent and youth-friendly health services in Ethiopia.

The finding of this study revealed that the overall magnitude of outcome dimensions of quality/client satisfaction was 57.01% (95% CI; 50.32, 63.7%) with a range from 47.2 (17) to 70.3% (30). A random effect model shows that heterogeneity was observed across the studies (I-squared = 94.7%, *p* < 0.001) (Figure 4).

##### 3.3.3.1. Sub-group analysis

As a result of heterogeneity in the satisfaction dimension of quality, we performed a subgroup analysis based on region, and publication year. In this regard, the magnitude of satisfaction was higher in the Oromia region studies (68.82%), and in studies conducted after 2020 (58.73%) when compared to SNNP studies and conducted before 2020 (Table 2).

**Table 2:** Subgroup analysis for the level of client's satisfaction with adolescent and youth-friendly health services in Ethiopia.

**Groups**	**Number of studies**	**Prevalence rate (95% CI)**	***I*^2^ (*P*-value)**
**Region**			
SNNP	2	50.23 (46.38–54.08)	0.00% (*P =* 0.46)
Amhara	4	53.89 (47.612–60.16)	87.7% (*P < * 0.001)
Oromia	2	68.82 (66.19–71.44)	0.00% (*P =* 0.404)
**Publication year**			
< 2020	4	55.24 (49.69–60.79)	77.4% (*P =* 0.004)
>2020	4	58.73 (47.06–70.41)	97.4% (*P < * 0.001)

##### 3.3.3.2. Sensitivity analysis

**Figure 5:**
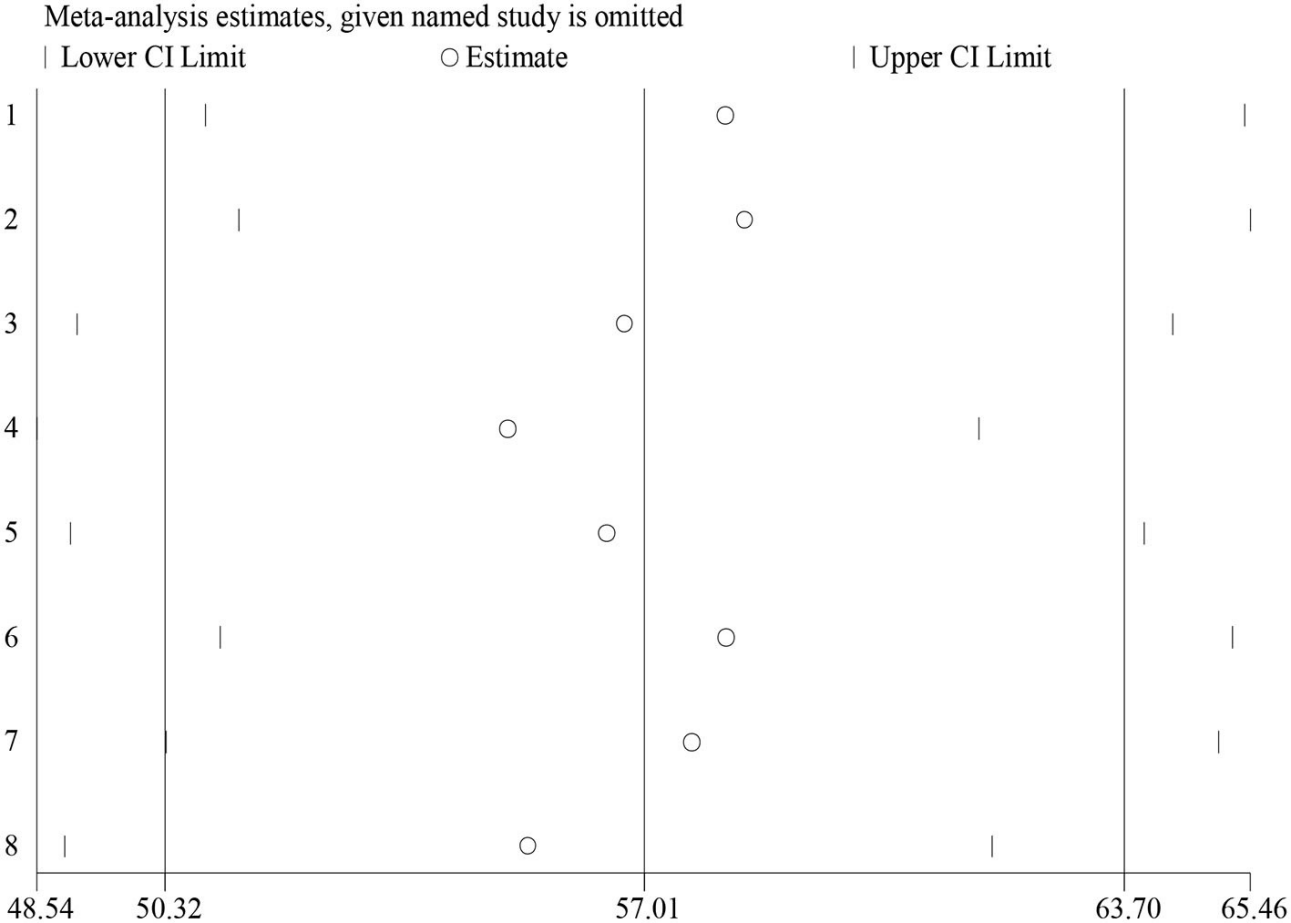
Sensitivity analysis of client's satisfaction with adolescent and youth-friendly health services in Ethiopia.

Sensitivity analysis is performed using a random-effects model to examine the contribution of each of the included studies to the final meta-analysis result. The result revealed that no single study affects the pooled level of client's satisfaction with adolescent and youth-friendly sexual and reproductive health services (Figure 5).

##### 3.3.3.3. Publication bias

**Figure 6:**
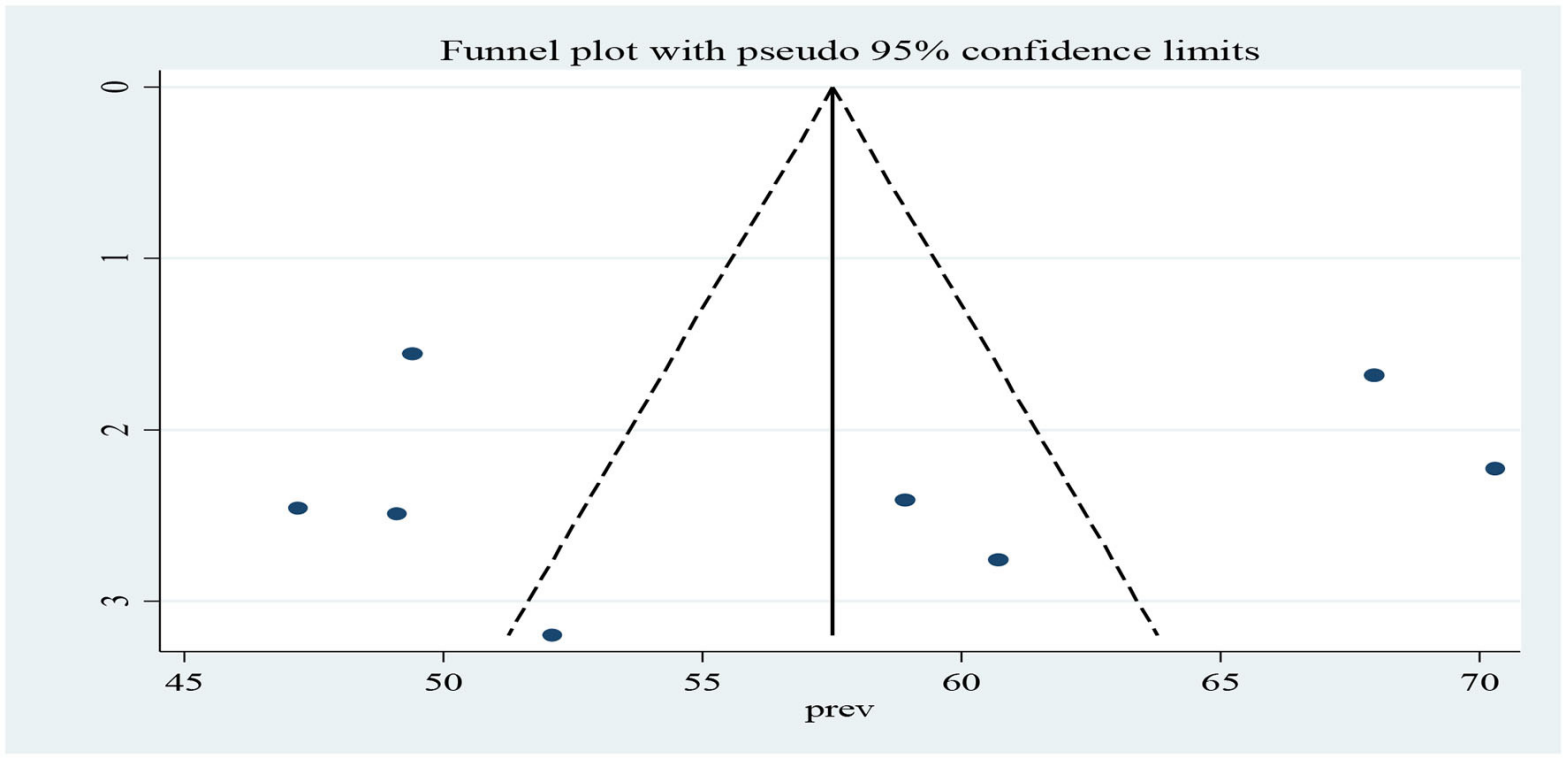
A funnel plot to demonstrate the publication bias of studies for the pooled the level of client's satisfaction with adolescent and youth-friendly health services in Ethiopia.

With respect to publication bias, it was assessed by using a funnel plot, which showed a symmetrical distribution of included studies (Figure 6). In addition, Begg's and Eggers's tests were checked and no significant publication bias observed among studies as evidenced by *p* = 0.0.805 and *p* = 0.804, respectively. Therefore, both methods revealed the absence of publication bias among studies.

### 3.4. Factors associated with client's satisfaction with YFSRH services

All authors have analyzed the potential socio-demographic and health service related factors influencing client's satisfaction with adolescent and youth-friendly health services in Ethiopia using data from eight studies. Variables that have been indicated at least in two studies were included in the meta-analysis. In those studies, socio-demographic variables such as sex, age, and employment status, and health service-related variables like length of waiting time to get services, comfort with service provider, get illness related services, information on the availability of services, the availability of services, and previous health services visit were included. Finally, four variables such as sex, employment status, waiting time to get services, and information on the availability of services were significantly associated with client's satisfaction with adolescent and youth-friendly health services (Table 3 and Supplementary material).

**Table 3:** A summary of factors associated with client's satisfaction with adolescent and youth-friendly sexual and reproductive health services in Ethiopia.

**Variables**	**Categories**	**OR (95%CI)**	***P*-value**
**Socio-demographic variables**			
Sex	Female	1.61 (1.14–2.27)	***p** **=*** **0.007**
	Male	1	
Age	10–19	0.52 (0.12–2.34)	*p =* 0.396
	20–24	1	
Employment status	Employed	1.82 (1.06–3.14)	***p** **=*** **0.031**
	Unemployed	1	
**Health service related variables**			
Length of waiting time to get services	≤ 30 min	2.7 (1.69–4.31)	***p** **=*** **0.000**
	>30 min	1	
Comfort with provider	Comfortable	1.14 (0.22–5.79)	*p =* 0.874
	Not comfortable	1	
Get illness related services	Yes	0.62 (0.33–1.16)	*p =* 0.132
	No	1	
Information on the availability of services	Informed	1.56 (1.15–2.11)	***p** **=*** **0.004**
	Not informed	1	
Availability of services	Yes	2.08 (0.52–8.39)	*p =* 0.304
	No	1	
Previous health services visit	Yes	2.19 (0.47–10.23)	*p =* 0.318
	No	1	

The bold values indicate that those independent variables with a bold *P*-value are significantly associated with the outcome variable.

Accordingly, female participants were 1.6 times more likely satisfied with adolescent and youth-friendly health services than males (AOR: 1.61, 95%CI: 1.14–2.27). Youths and adolescents who were employed were near to two times (AOR: 1.82, 95% CI: 1.06–3.14) as likely satisfied with provided services as their unemployed counterparts. In addition, respondents who took <30 min to get services and informed about the available services were around three times (AOR: 2.7, 95%CI: 1.69–4.31) and 1.6 times (AOR: 1.56, 95%CI: 1.15–2.11) more satisfied with provided services than their counterparts, respectively (Table 3 and Supplementary material).

## 4. Discussion

The current study assessed the pooled level of quality for adolescent and youth-friendly sexual and reproductive health services in Ethiopia. According to this meta-analysis, the national pooled magnitude of structural, process, and output dimensions of quality is 54.22% (95% CI: 33.21, 75.24%), 35.44% (95% CI: 24.95, 45.93%), and 57.01% (95% CI: 50.32, 63.7%), respectively. Although significant differences were observed between quality components, this is consistent with a study conducted in South Africa, in which the quality of AYFHS was 48% (36), which was below the WHO and the National Adolescent and Youth Health Strategy standards (the cutoff point for “good quality” is 75%) (27, 37).

In terms of structural components, it is comparable to the YFS assessments conducted in Uganda (12), but it is of lower or poorer quality when compared to South African (13) and WHO standards (27). This could be due to a lack of resources and competing health priorities in the study area.

Similarly, process indicators are consistent with findings in Uganda (12) and are not in line with or lower than the WHO quality standard set for YFS services (27, 38). This may be the result of differences in the health care system related to insufficient provider training, competency, and specialization.

Furthermore, overall client satisfaction with AYFHS was lower than in a study conducted in Tanzania (14), South Africa (13) and Switzerland (15), in which 89, 87.1, and 94% of adolescents were satisfied with services, respectively. The possible explanation for the discrepancy between the two African studies could be sociocultural differences or differences in the health service delivery system. The difference with the Switzerland study also could be due to the difference in sex of the study participants; both sexes were included in this study, but only females were studied in the previous one. Subjective satisfaction ratings and AYFHS client expectations may both play a role in this discrepancy.

Female participants were 1.6 times more likely to be satisfied with adolescent and youth-friendly health services than male participants. This finding is in line with a study across four West African countries (20) and the European regions (22). The possible explanation could be that females are more utilized by the service than males, and when they become clients, client could understand the service better than the newcomer, which may deviate the rate of satisfaction for the female.

Youths and adolescents who were employed were nearly two times more satisfied with services than their counterparts. This finding is supported by a study done in Serbia (21), Kerman hospitals, Iran (18). It might be due to the fact that unemployed patients tend to estimate their health conditions as worse and are preoccupied with the perception that the service quality provided to them might be poor, which may create a communication barrier with health workers. However, this study contradicts a study conducted in America that found that employed clients tend to have lower satisfaction than their counterparts (19). This difference could be due to differences in client attitude, provider practice, and expectations of service quality.

In addition, clients who took <30 min to get services were around three times more satisfied with the provided services than their counterparts. This result is supported by the WHO service quality standards, in which decreasing clients waiting times to get services is one of the strategies (3, 27). The finding is not consistent with the study conducted in China (39). This may be attributed to the low service utilization, the proportional number of health care providers with clients, and the participants' awareness that some health care services require time to provide quality care.

Furthermore, clients who got information about the available services were 1.6 times more satisfied with the provided services than their counterparts. This finding is supported by global and national quality service standards (24, 27). This is in line with the truth that information is crucial to utilizing the services and for the clients to be satisfied at first.

## 5. Limitations of the study

Since only English search terms were used in the search strategy and papers without an English abstract were not considered for inclusion in the review, it is doubtful that this review has found all pertinent studies. Moreover, we did not search all of the gray literature. The ability to compare the findings of different research may be hampered by differences in quality criteria, measuring techniques, and study setting.

## 6. Conclusions

This review result shows that the national pooled magnitude of structural, process, and output dimensions of the quality of adolescent and youth-friendly sexual and reproductive health services is far below WHO quality of services standards, which are 75 percent for good quality.

Variables such as sex, employment status, waiting time to get services, and information on the availability of services were significantly associated with client satisfaction with adolescent and youth-friendly health services.

The FMOH, RHB, Zonal Health Department, Woreda Health Office, NGOs, and other responsible bodies should work together to strengthen the quality of adolescent and youth sexual and reproductive health services. The structural inputs of the health facilities need improvement, and the interaction between providers and clients should be strengthened so that service users are satisfied with the services that you are going to provide. Longer wait times for service shall be reduced by allocating an adequate number of providers and expanding service delivery points to make service more accessible. Furthermore, clients shall be made aware of the nature or timing of the services they have requested, and the health care providers should comply with the national guidelines. In addition, providing adequate information to the service users on the availability of services using different information-education-communication media, like radio, may improve the quality of services. Furthermore, observational, qualitative, and mixed types of studies are required to provide comprehensive evidences and they should focus on investigating the impact of these factors on other health outcomes and exploring additional factors that influence the quality of adolescent and youth-friendly health services.

## Data availability statement

The original contributions presented in the study are included in the article/Supplementary material, further inquiries can be directed to the corresponding author.

## Author contributions

GAZ, TME, MMB, and AMA performed the literature search, data extraction, data analysis, wrote the manuscript, and reviewed and revised the paper. All authors reviewed and approved the final manuscript.
