# Supplementary material for: Quality of adolescent and youth-friendly sexual and reproductive health services and associated factors in Ethiopia: a systematic review and meta-analysis
Source: Front Public Health. 2023 Jul 12;11:1191676. doi: 10.3389/fpubh.2023.1191676 (PMC10369062; doi:10.3389/fpubh.2023.1191676)
Supplement: Supplementary file 1 [file Presentation_1.pdf]

Appendix: Supplementary Figures

Appendix I: Factors associated with satisfaction with health services

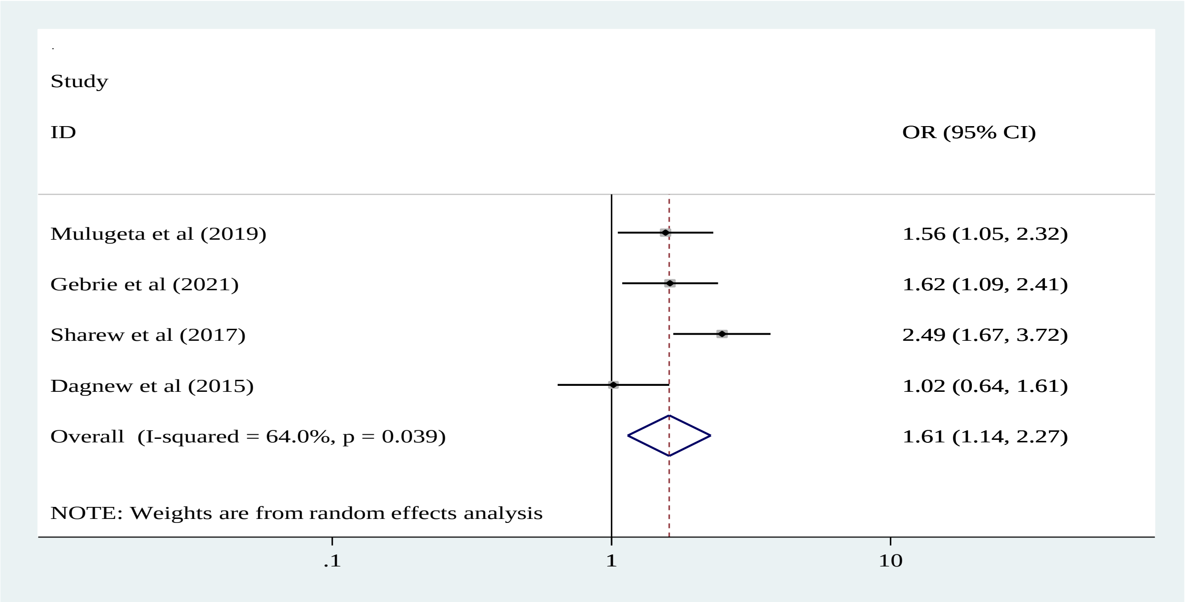

Figure 7: The association between participants' sex and satisfaction with adolescent and youth-friendly sexual and reproductive health services in Ethiopia

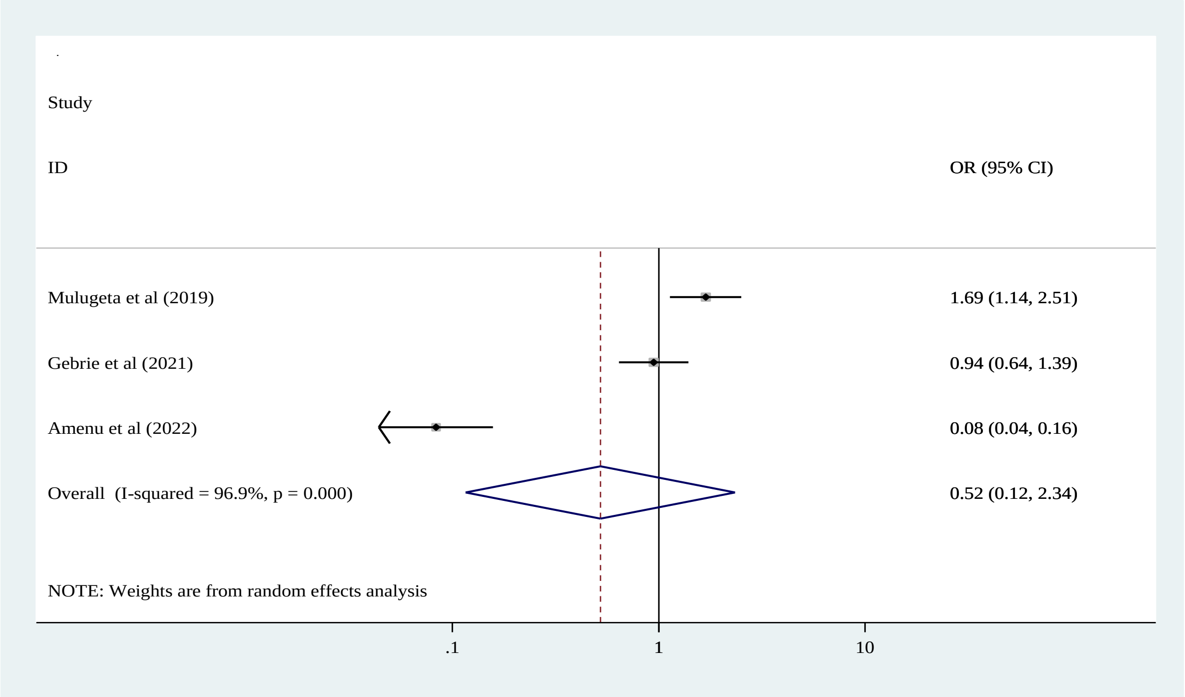

Figure 8: The association between participants' age and satisfaction with adolescent and youth-friendly sexual and reproductive health services in Ethiopia

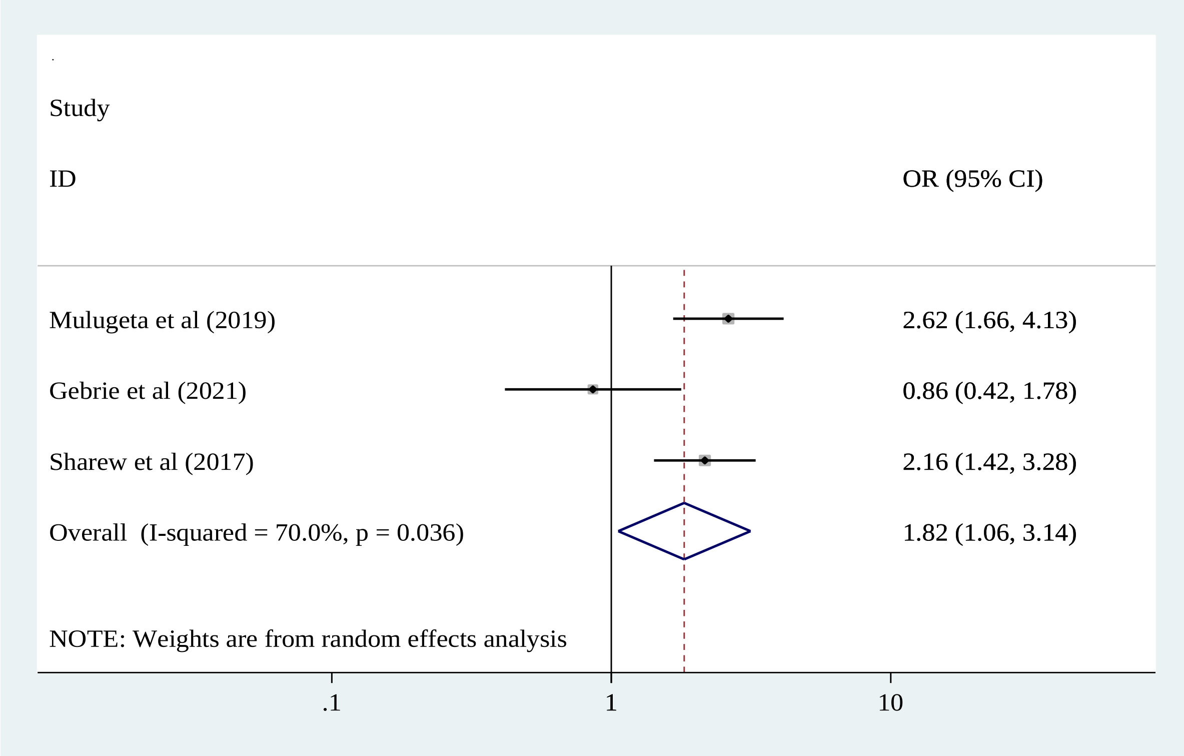

Figure 9: The association between participants' employment status and satisfaction with adolescent and youth-friendly sexual and reproductive health services in Ethiopia

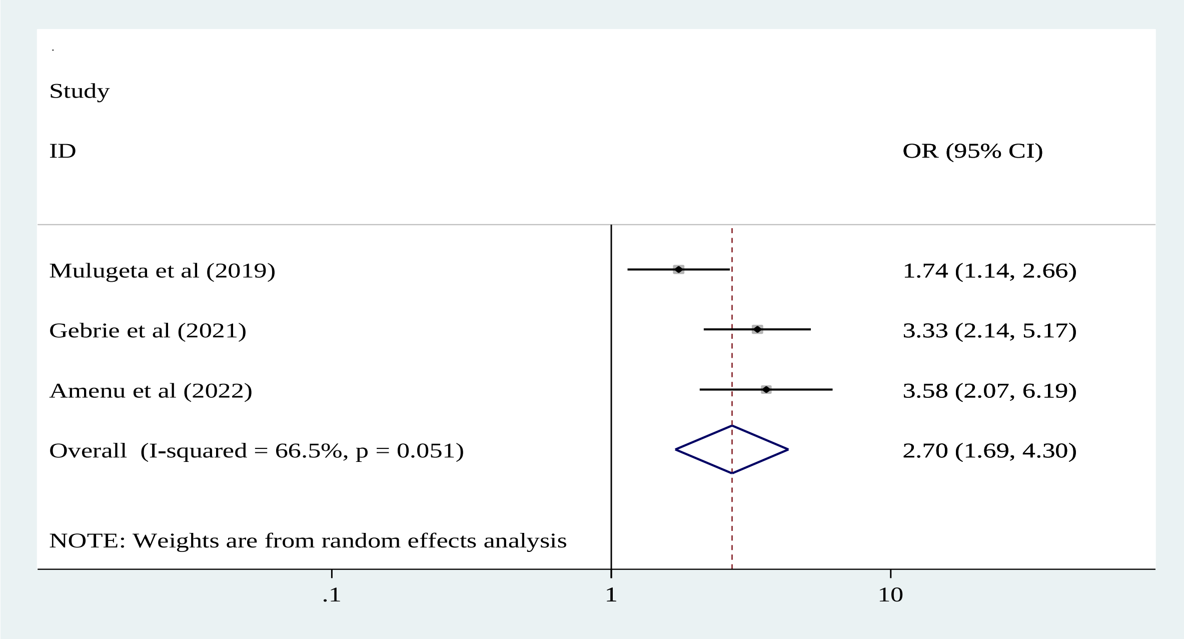

Figure 10: The association between waiting time to get services and satisfaction with adolescent and youth-friendly sexual and reproductive health services in Ethiopia

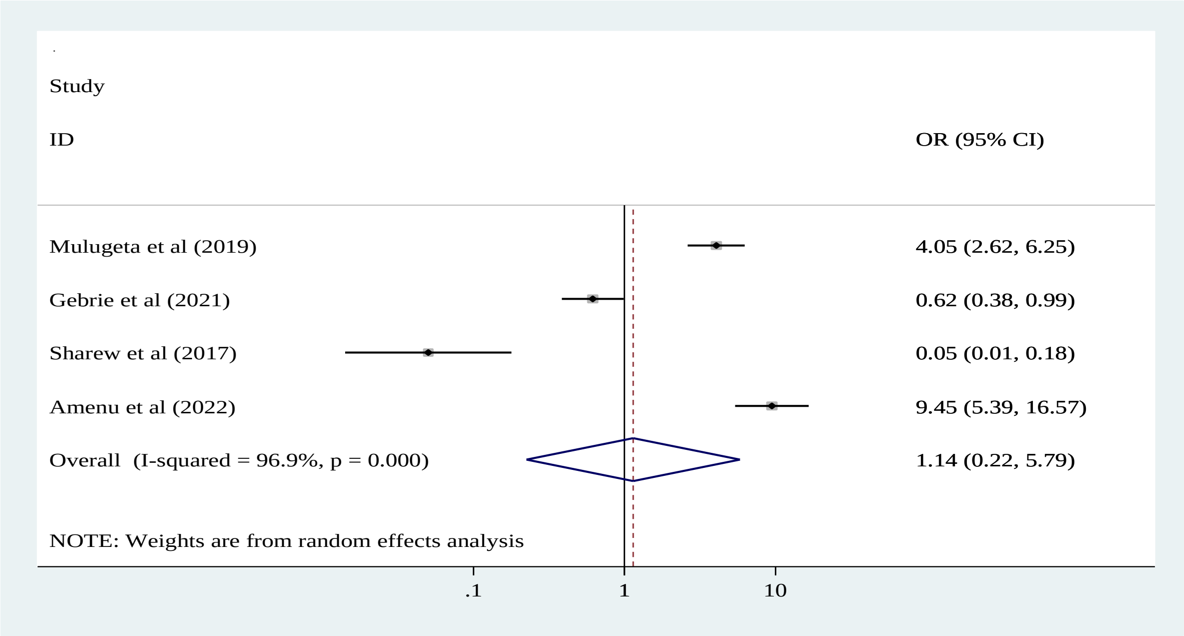

Figure 11: The association between clients' comfort with service providers and satisfaction with adolescent and youth-friendly sexual and reproductive health services in Ethiopia

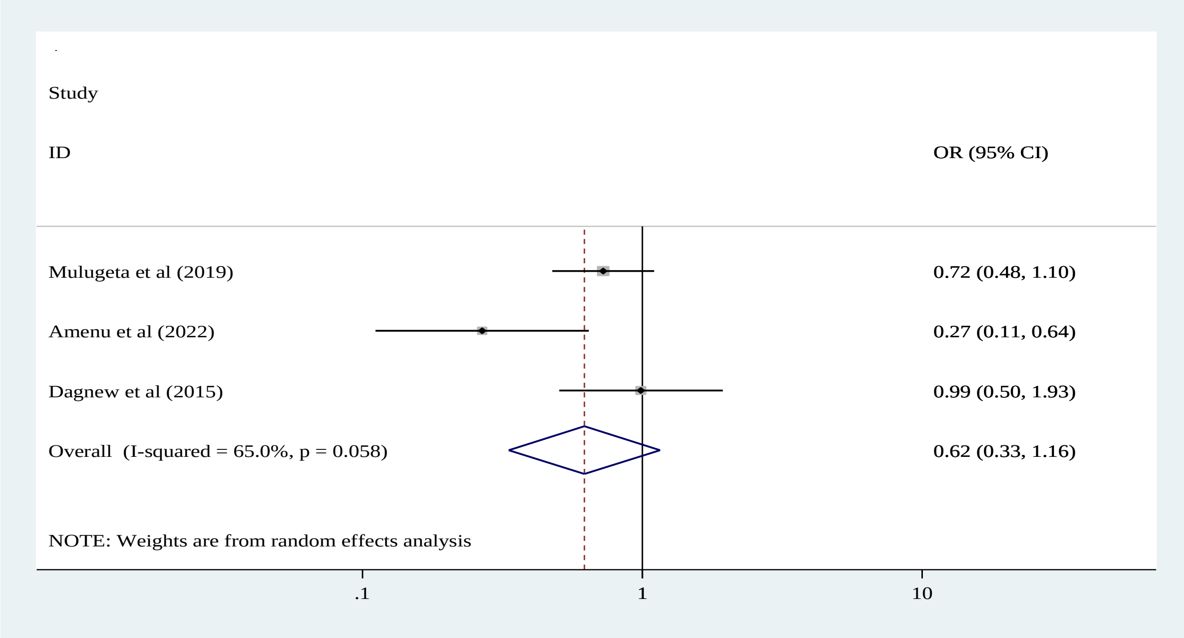

Figure 12: The association between the availability of illness-related services and satisfaction with adolescent and youth-friendly sexual and reproductive health services in Ethiopia

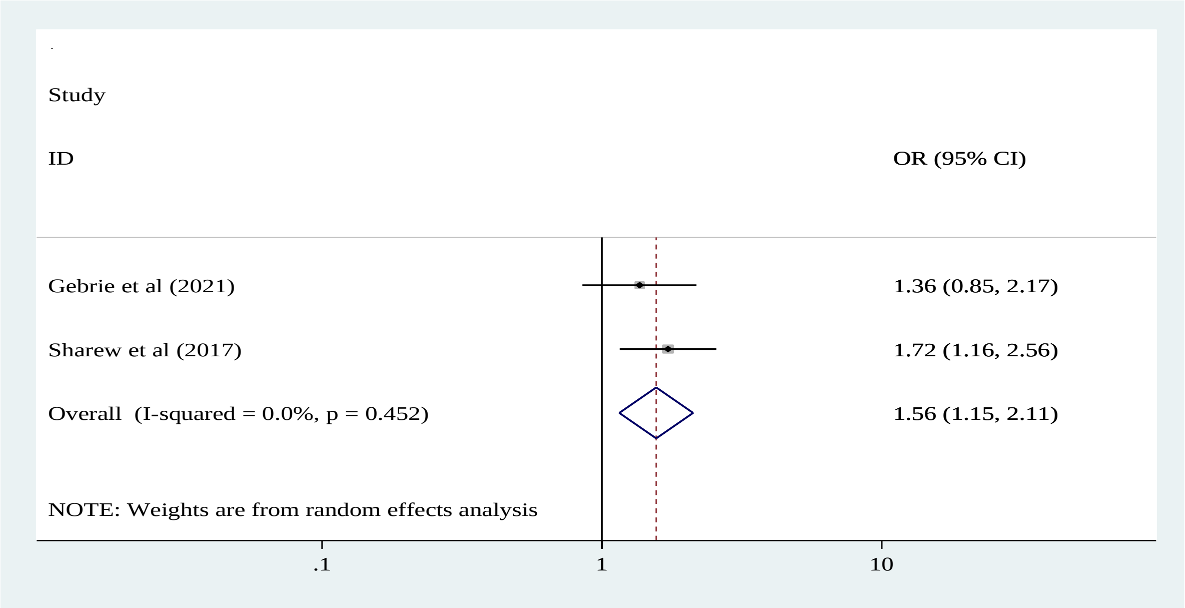

Figure 13: The association between the availability of information on providing services and satisfaction with adolescent and youth-friendly sexual and reproductive health services in Ethiopia

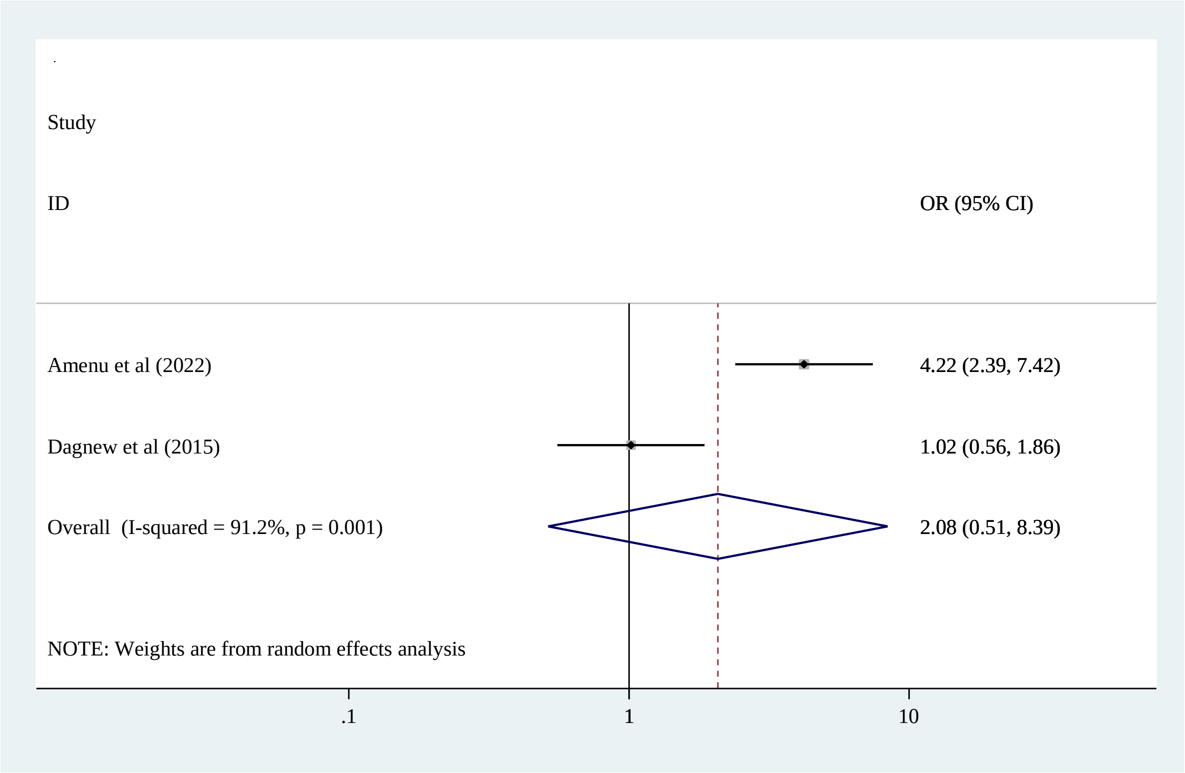

Figure 14: The association between the availability of services and satisfaction with adolescent and youth-friendly sexual and reproductive health services in Ethiopia

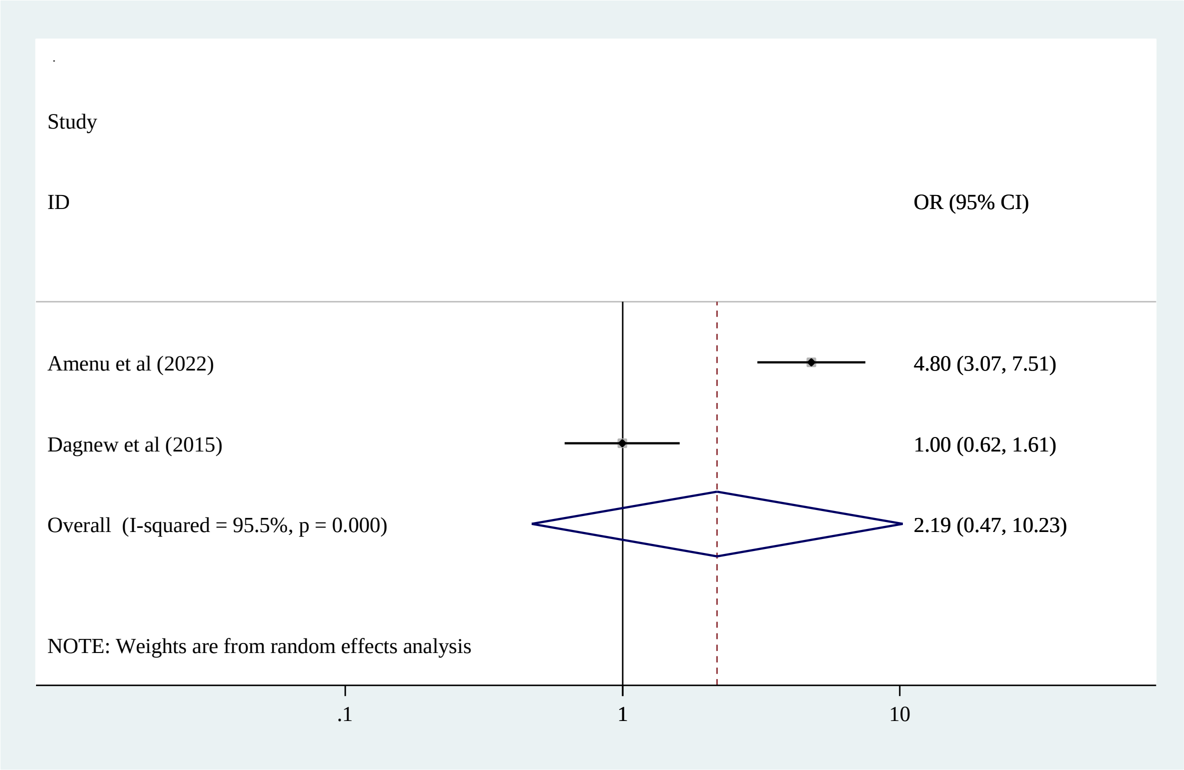

Figure 15: The association between previous health services visit and satisfaction with adolescent and youth-friendly sexual and reproductive health services in Ethiopia
